# Cholecystocolonic fistula: A rare case report of Mirizzi syndrome^☆^

**DOI:** 10.1016/j.ijscr.2019.09.023

**Published:** 2019-09-24

**Authors:** C.A. Esparza Monzavi, X. Peters, M. Spaggiari

**Affiliations:** a Department of General Surgery, University of Illinois Hospital at Chicago, Chicago, IL, USA; b College of Medicine, University of Illinois at Chicago, Chicago, IL, USA; c Division of Transplantation, Department of Surgery, University of Illinois at Chicago, Chicago, IL, USA

**Keywords:** Case report, Mirizzi syndrome, Cholecystocolonic fistula, Cholecystoenteric fistula

## Abstract

**Introduction:**

Mirizzi syndrome is a rare complication of gallstone disease that more rarely is associated with the formation of cholecystoenteric fistula.

**Presentation of case:**

The patient presented with a five-day history of abdominal pain in the right upper quadrant (RUQ), nausea, and emesis. Further ultrasound (US) imaging demonstrated a large gallstone with associated thickened gallbladder with pericholecystic fluid. Computed tomography (CT) imaging, preoperative Hepatobiliary Scintigraphy and Endoscopic Retrograde Cholangiopancreatography (ERCP) displayed findings consistent with a Csendes type IV Mirizzi syndrome associated with cholecystocolonic fistula. Description of surgical approach, management and outcome is presented.

**Discussion:**

Surgical management of Mirizzi syndrome varies by classification of its severity. Open operation is preferred in cases with severe inflammation and concern for malignancy. The patient underwent a cholecystocolonic fistula takedown. A cholecystectomy was attempted though aborted due to concerns of malignancy. Biopsies returned negative for malignancy and the patient demonstrated findings on ERCP consistent with Mirizzi syndrome.

Stenting of the common bile duct (CBD) was performed with ERCP and later the patient underwent an open biliary exploration with subsequent choledochotomy, biliary stone removal, and primary closure with interrupted sutures using remnant gallbladder wall flaps.

**Conclusion:**

To our knowledge, Mirizzi syndrome with concurrent cholecystocolonic fistula is exceedingly rare with a paucity of reports within the literature. Our report discusses principles of management of Mirizzi syndrome as well as best practices of surgical management for Mirizzi syndrome with concurrent cholecystocolonic fistula.

## Highlights

• Mirzzi syndrome is a rare complication of gallstone disease that involves compression on the biliary tree by a stone within the gallbladder.
• Rarely, in advanced disease Mirizzi syndrome may present with concurrent choleystoenteric fistula.
• Cholecystocolonic fistula are rare in the setting of Mirizzi syndrome.
• In Mirizzi syndrome with cholecystocolonic fistula, possible malignancy in the setting of severe inflammation must be considered.

## 1 Introduction

Mirizzi syndrome, eponymously documented in 1948, is now widely known amongst the surgical community to denote the circumstance by which a large gallstone in the gallbladder neck or cystic duct leads to a narrowing of the common hepatic duct [1]. Mirizzi syndrome is among the rarer complications of longstanding gallstone disease, alongside cholecystocholedochal fistula and gallstone ileus [2]. Mirizzi syndrome concurrent with cholecystoenteric fistula is an even rarer occurrence and may or may not include an associated gallstone ileus [2,3]. Of those patients found to have both Mirizzi syndrome as well as cholecystoeneteric fistula, reports have included cholecystoduodenal fistula as well as cholecystogastric fistula. However, there are few reports of cholecystocolonic fistula [2]. In the following case, the patient was discovered to have advanced Csendes type V Mirizzi with a cholecystocolonic fistula, representing a rare phenomenon with a rarer presentation. This phenomenon has been explained in detail in only one prior case at the time of this writing [4]. This case was handled at an academic institution and has been presented in accordance with the SCARE criteria [5].

## 2 Presentation of case

A 70-year old man with history of hypertension, hyperlipidemia, and chronic biliary colic presented to an outside medical center with a five day history of RUQ abdominal pain, nausea and emesis that worsened with fatty meals. The patient denied fevers or diarrhea, was afebrile, and vital signs were within normal limits. On exam, the patient demonstrated RUQ and epigastric tenderness with a negative murphy sign. Initial laboratory tests were notable for an alkaline phosphatase of 183 and were otherwise unremarkable.

**Fig. 1:**
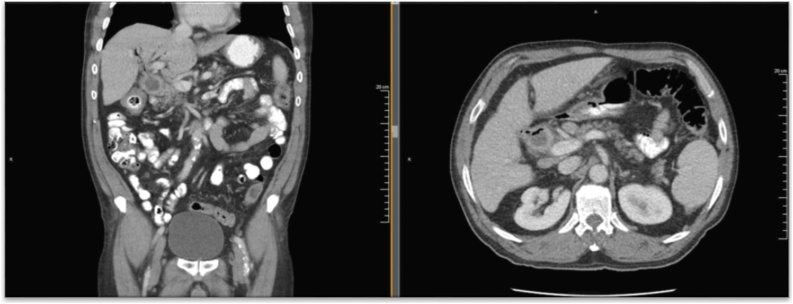
a. coronal section of abdominal CT scan demonstrating thickening extending from the gallbladder to the hepatic flexure of the colon b. transverse section of findings described in 1a.

**Fig. 2:**
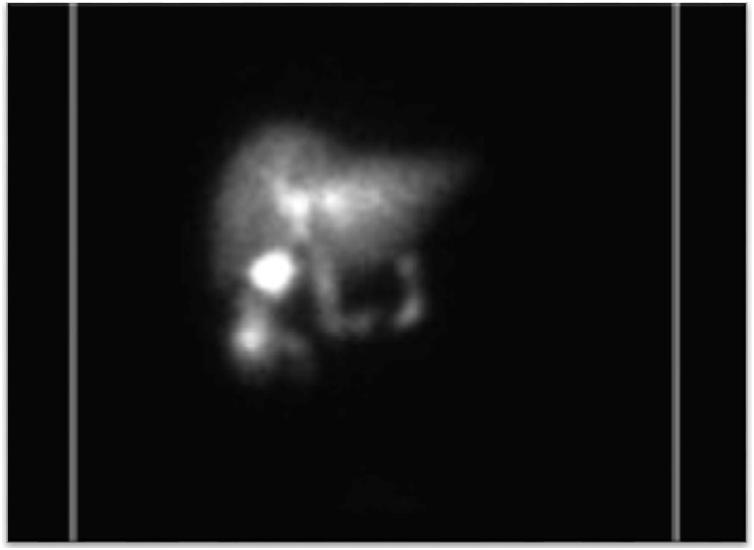
Preoperative Hepatic Scintigraphy suggestive of biliary flow to the colon through cholecystocolonic fistula in addition to its trajectory through the biliary tree.

Ultrasound (US) imaging demonstrated a thickened gallbladder wall up to 5 mm with an apparent shadowing suggesting a gallstone as well as some pericholecystic fluid. Due to extensive ring-down artifact on US, computed tomography (CT) imaging was obtained and elicited concern for cholecystocolonic fistula (Fig. 1); with pneumobilia, inflammation and enhancement in the area of the gallbladder fossa and common bile duct (CBD), as well as thickening extending into the adjacent colon at the hepatic flexure. The patient underwent two weeks of antibiotic therapy. Preoperative Hepatobiliary scintigraphy was compatible with a cholecystocolonic fistula to the hepatic flexure of the colon (Fig. 2). However, preoperative colonoscopy was unable to locate the fistula tract.

On an elective basis, the patient was taken to the operating room. After laparoscopic exploration, the decision was made to convert to open due to signs of severe inflammation. An open cholecystocolonic fistula takedown with partial colectomy and primary anastomosis was performed. A cholecystectomy was attempted though aborted due to concern for malignancy and biopsies were taken. The patient was transferred to our institution for further care. At the time of transfer, the patient was hemodynamically stable and tolerating diet without complaint. There was a kocher incision healing well and a single Jackson Pratt (JP) drain with mild bilious drainage. Laboratory tests were notable for a slightly elevated bilirubin to 1.3 and an albumin of 2.9. Biopsies taken at the outside institution were found to be negative.

**Fig. 3:**
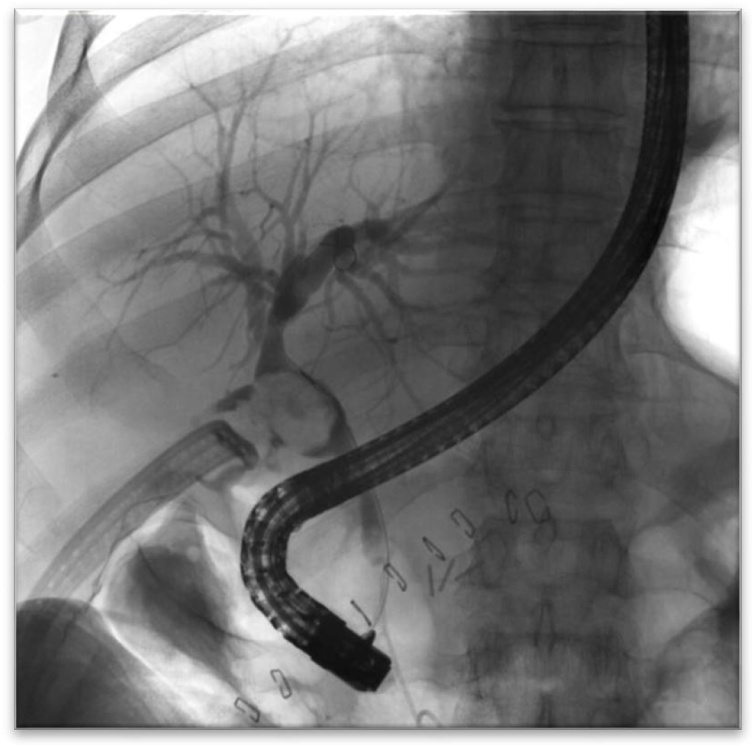
ERCP indicating multiple filling defects in the hepatic ducts and suggestive of a large stone eroding through the wall of the upper third of the common hepatic duct, concerning for Mirizzi type IV.

**Fig. 4:**
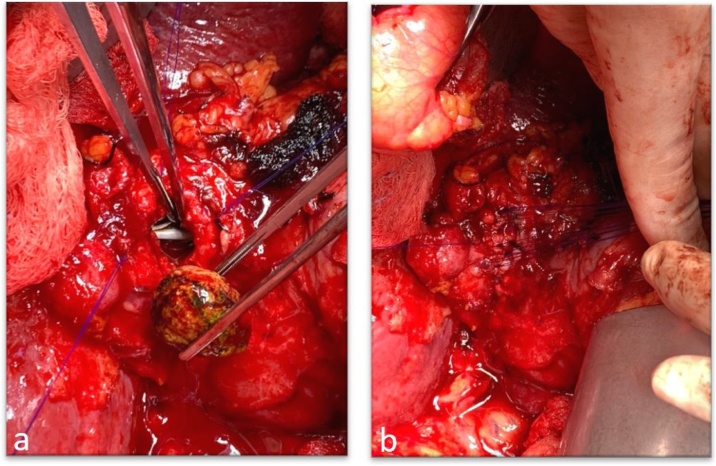
Intraoperative photos during exploratory laparotomy and bile duct exploration with choledochotomy. a. choledochotomy revealing large stone previously eroding through the upper one-third of the common hepatic duct. b. gallbladder remnant repaired following choledochotomy.

The patient underwent a diagnostic ERCP and was found to have multiple stones in the hepatic ducts as well as a large stone eroding through the wall of the upper third of the common hepatic duct concerning for Csendes type IV Mirizzi syndrome (Fig. 3); stent was placed in the CBD. Following complete preoprative planning, the patient underwent an exploratory laparotomy, bile duct exploration with a choledochotomy and removal of a large biliary stone. The stent was visible in the lumen of the bile duct (Fig. 4a) and the remainder of the gallbladder neck was used to primarily repair the defect using interrupted sutures (Fig. 4b).

The patient tolerated the procedure well, nasogastric tube (NGT) and Foley catheter were removed on postoperative day (POD) 1. His diet was advanced and he was tolerating solid food as of POD2. On POD 3 the output from JP drain was minimal and it was removed. He was discharged home in good condition on POD4 and later seen in clinic with a well healed scar and no other complaints

## 3 Discussion

Through the decades since its discovery, Mirizzi syndrome has presented with various levels of severity and effect, necessitating multiple classification schemes. Ultimately, those most widely used in clinical practice include the McSherry and the Csendes classification systems [1,6]. The McSherry classification schema dichotomized the syndrome, denoting type I as external compression of the bile duct by a large stone, and type II as a cholecystobiliary fistula caused by such a stone or stones [7]. The Csendes classification provides additional utility, as it correlates with principles of management. This formulation delineated severity of damage to the biliary tree, where Csendes type II indicates a cholecystobiliary fistula with less than one-third circumference of the biliary tree eroded. Further, types III and IV indicate erosion of up to two-thirds, and erosion resulting in complete destruction of the duct, respectively. Csendes type V is indicative of a cholecystoenteric fistula in addition to any of the previously stated types. This is further divided into patients without a gallstone ileus, known as type Va, and those with an associated gallstone ileus, known as type Vb [3].

While Ultrasound is widely used and is recommended as the best screening method, no significant difference has been demonstrated in the sensitivity of detection between groups with cholecystobiliary fistula and those without [8]. Magnetic Resonance Cholangiopancreatography (MRCP) may be more advantageous, as it can further delineate pericholecystic inflammation as well as help to differentiate Mirizzi syndrome from gallbladder malignancy. ERCP remains the gold standard of diagnosis, though the full details of its presentation in individual patients may not be recognizable until operative intervention [8].

Notable variants of Csendes type V Mirizzi syndrome have been described, including Mirizzi combined with Bouveret syndrome [9]. Though cases have been reported, the occurrence of Mirizzi syndrome with a concurrent cholecystoenteric fistula remains a rare presentation of this disease. The occurrence of cholecystoenteric fistula in patients with Mirizzi has been shown to correlate to the severity of Mirizzi described by progression from Csendes type I to IV [3]. Further, the occurrence of Mirizzi syndrome with concurrent cholecystocolic fistula is rarer yet [4]. Because the surgical approach varies based on severity, it is important to stage the procedure appropriately.

Surgical management of Mirizzi varies by Csendes type. Cholecystectomy and partial cholecystectomy have been shown to be effective for Csendes type I. The approach to type II and III involve partial cholecystectomy without removal of tissue at the margin of the fistula. Management of severe, Csendes type IV may warrant a Roux-en-Y hepaticojejunostomy [3,8]. Open operation is preferred with severe inflammation within Calot’s triange in the setting of distorted anatomy and is the current standard of management for Mirizzi syndrome [8]. Despite the staged, multicenter care this patient received, it is the position of the authors that a case like this is best managed in a single operation to minimize the risk of seeding potential malignancy. Concern for gallbladder malignancy would best be managed in this scenario by frozen section, as this has been demonstrated to be an effective method for identifying lesions that would necessitate conversion to radical surgery [10].

Our patient had biopsies negative for malignancy. Due to the large defect of the common biliary duct, reconstruction of the duct was made using remnant walls of the gallbladder neck using interrupted stiches, leaving the stent in place (Fig. 4).

## 4 Conclusion

To our knowledge, this is one of two cases within the literature describing a patient with Mirizzi syndrome concurrent with cholecystocolonic fistula [4]. This case not only highlights the rare possibilities of anatomical presentation in advanced gallstone disease but delineates our surgical management. Despite the multicenter management of the patient described, a single surgical endeavor would be better suited for a patient with such advanced gallbladder disease with concern for malignancy. We aim to present this case as another example of a rare presentation of Mirizzi syndrome that, due to its rarity, heretofor lacks well document standards of managment.

## Funding

No source of funding to be declared.

## Ethical approval

No institutional review board is required for publication of a case report at our institution.

## Consent

Written informed consent was obtained from the patient for publication of this case report and its accompanying images.

## Author’s contribution

Esparza Monzavi, CA – study concept/design, data interpretation, writing the paper.

Peters, XD – data interpretation, writing the paper.

Spaggiari, M – study concept/design, data interpretation.

## Registration of research studies

NA.

## Guarantor

Spaggiari, M.

Esparza Monzavi, CA.

## Provenance and peer review

Not commissioned, externally peer-reviewed.

## Declaration of Competing Interest

The authors declare no conflicts of interest pertinent to this case report.
